# A novel *de novo ATP2B1* variant causes autosomal dominant intellectual developmental disorder 66 by disrupting calcium homeostasis via impaired membrane trafficking

**DOI:** 10.3389/ebm.2026.10834

**Published:** 2026-03-03

**Authors:** Huanhuan Zang, Xiaoyun Yang, Yucai Liu, Caiyun Ma, Dawei Yang

**Affiliations:** 1 Department of Pediatrics, The First Affiliated Hospital of Bengbu Medical University, Bengbu, China; 2 Anhui Engineering Research Centre for Neural Regeneration Technology and Medical New Materials, Bengbu Medical University, Bengbu, Anhui, China; 3 Department of Rheumatology and Immunology, Bengbu Hospital of Shanghai General Hospital/The Second Affiliated Hospital of Bengbu Medical University, Bengbu, China

**Keywords:** *ATP2B1*, calcium homeostasis, *de novo* variant, intellectual developmental disorder 66, neurodevelopmental disorder

## Abstract

Heterozygous pathogenic variants in *ATP2B1* (encoding PMCA1) cause autosomal dominant intellectual developmental disorder 66 (MRD66; OMIM #619910). To date, only 12 pathogenic *de novo ATP2B1* variants have been reported in MRD66. This study aimed to identify the genetic etiology in a Chinese infant with a neurodevelopmental disorder characterized by early-onset seizures and global developmental delay (GDD) and functionally characterize a novel *ATP2B1* missense variant. Trio-based whole-exome sequencing revealed a heterozygous *de novo ATP2B1* variant (c.2140A>C, p.Thr714Pro) in the proband. The proband presented with infantile spasms, GDD (Gesell Developmental Quotient: 65–74), and severe growth restriction (height/weight <−2 SD). To investigate the variant’s pathogenicity, the wild-type (WT) and mutant *ATP2B1* constructs, N-terminally tagged with mScarlet, were transfected into HEK293T cells. Confocal imaging demonstrated profound cytoplasmic mislocalization of the p.Thr714Pro mutant protein, contrasting sharply with the characteristic plasma membrane localization of WT ATP2B1. Measurement of intracellular Ca^2+^ levels using Fluo-4 AM showed a significant 2.07-fold increase in basal Ca^2+^ levels in cells expressing the mutant compared to WT. This finding expands the spectrum of ATP2B1 variants associated with MRD66 and confirms calcium dyshomeostasis as the core pathomechanism. This case of MRD66 demonstrates a very early onset of seizures, consistent with the recognized phenotypic variability and the critical role of PMCA1 in early neurodevelopment.

## Impact statement

This manuscript contributes significantly to understanding genetic causes of early-onset epileptic encephalopathy and developmental delay. It identifies a novel ATP2B1 variant in a patient with severe developmental impairment and infantile spasms, demonstrating how this genetic change disrupts calcium balance in cells through two mechanisms: improper protein localization and reduced calcium export. This work expands the spectrum of known ATP2B1 variants linked to neurodevelopmental disorders and highlights the critical role of calcium regulation in early brain development and function. By providing new insights into the genetic architecture and pathophysiology of these conditions, the study advances diagnostic approaches and points toward potential therapeutic strategies targeting calcium homeostasis. The findings also underscore the variability in clinical presentation associated with ATP2B1 variants, offering valuable information for genetic counseling and clinical management.

## Introduction

Neurodevelopmental disorders (NDDs) with early-onset seizures and associated developmental delay represent a genetically heterogeneous group of neurodevelopmental disorders (NDDs) [1]. While environmental factors like gestational infections or teratogen exposure can contribute, pathogenic *de novo* variants (DNMs) in critical developmental genes are a major etiology, particularly in undiagnosed cases, roughly divided equally between loss-of-function (LoF) and altered-function variants, which are often missense and can confer gain-of-function, dominant-negative, or other pathogenic mechanisms [2]. Disrupted intracellular Ca^2+^ signaling has emerged as a key pathological mechanism underlying both neurodevelopmental deficits and epilepsy.

Calcium ions (Ca^2+^) function as universal second messengers essential for fundamental neuronal processes. They regulate diverse functions including signal transduction cascades, gene expression, cellular metabolism, neurotransmitter release, synaptic plasticity, and membrane excitability [3]. Within neurons, precise Ca^2+^ homeostasis is critical for a suite of processes indispensable for learning and memory, such as neurotransmitter release, neurite outgrowth, synaptogenesis, and synaptic efficacy. Dysregulation of Ca^2+^ signaling is implicated in neurodegeneration (e.g., Alzheimer’s, Parkinson’s, Huntington’s diseases) and is a well-established driver of epileptogenesis. Pathogenic variants in voltage-gated calcium channel genes, such as *CACNA1H* which encodes T-type channels, can lower activation thresholds and prolong channel opening, causing pathological Ca^2+^ influx, neuronal hyperexcitability, and seizures [4]. Elevated intracellular Ca^2+^ potentiates presynaptic glutamate release [5], while simultaneously desensitizing postsynaptic GABAergic receptors, thereby impairing inhibitory neurotransmission despite normal GABA levels [6]. This dual action disrupts the critical excitatory-inhibitory (E/I) balance within neuronal networks, facilitating seizure initiation and propagation. Clinically, the efficacy of Ca^2+^ channel-targeting antiepileptic drugs (e.g., ethosuximide, zonisamide) provides strong validation for the proposed pathomechanism of calcium dyshomeostasis [7].

Plasma membrane Ca^2+^-ATPase type 1 (PMCA1), encoded by the *ATP2B1* gene (OMIM #108731, Chr12q21.33), is a P-type ATPase crucial for maintaining low resting cytosolic Ca^2+^ levels. It actively exports Ca^2+^ against its steep concentration gradient, utilizing energy derived from ATP hydrolysis, forming a characteristic aspartyl phosphate intermediate during its catalytic cycle [8]. PMCA1 belongs to a family of four calmodulin-dependent Ca^2+^ pumps in mammals (*ATP2B1*, *ATP2B2* [Chr3p26], *ATP2B3* [ChrXq28], *ATP2B4* [Chr1q25]). Functional diversity within this family is further expanded by extensive alternative splicing and tissue-specific expression patterns (PMCA1 is ubiquitously expressed, with high levels in the brain and bone marrow). The 1,220-amino acid PMCA1 protein features a C-terminal calmodulin-binding autoinhibitory domain and contains consensus sites for phosphorylation by cAMP-dependent kinase. Beyond its primary role in calcium extrusion, PMCA1 regulates insulin sensitivity in endothelial cells through Ca^2+^/calmodulin-dependent activation of AKT1 and NOS3 signaling pathways [9]. The embryonic lethality observed in *Atp2b1* homozygous knockout mice underscores its essential non-redundant role in development [10].

Autosomal dominant intellectual developmental disorder 66 (MRD66; OMIM#619910) is caused by heterozygous pathogenic *de novo* variants in *ATP2B1*. Characterized by variable expressivity, the core phenotypic features include global developmental delay (GDD), mild-to-moderate intellectual disability (ID), and seizures, which occur in ∼50% of patients. The other accompanying features include autism spectrum disorder (ASD), subtle dysmorphic features, and others. To date, only 12 distinct pathogenic *de novo ATP2B1* variants have been conclusively associated with MRD66 [8], limiting the understanding of robust genotype-phenotype correlations. This knowledge gap underscores the critical importance of functional validation for novel variants to elucidate their specific pathomechanisms.

In the current study, we report an infant with a neurodevelopmental disorder featuring infantile spasms and GDD harboring a novel *de novo ATP2B1* missense variant (p.Thr714Pro). Structural modeling predicts that this variant disrupts critical hydrogen bonding within the catalytic P-domain. We then employed confocal microscopy and live-cell calcium imaging to functionally validate that this alteration causes PMCA1 mislocalization away from the plasma membrane and ablates Ca2+ efflux capacity, leading to cytotoxic calcium overload. These findings significantly expand the mutational spectrum of MRD66 and provide direct mechanistic evidence implicating calcium dyshomeostasis as the central neuropathogenic process.

## Materials and methods

### Patient recruitment

We recruited a proband with epilepsy from non-consanguineous parents. The legal guardian of the proband provided informed consent and was aware of the anonymous publication of clinical and genetic information of the proband. The medical ethics of this study have been approved by the Institutional Review Board of the First Affiliated Hospital of Bengbu Medical University (ID: 2025049).

### Genetic analysis

Genomic DNA was extracted from peripheral blood lymphocytes of the proband and both parents using a Blood Genomic DNA Extraction Kit (CWBIO Biotechnology). DNA concentration and quality were assessed using a Qubit^®^ 3.0 Fluorometer (Thermo Fisher Scientific). Whole-exome sequencing (WES) libraries were prepared using the IDT xGen Exome Research Panel v2.0 and sequenced on an Illumina NovaSeq 6000 platform (2 × 150 bp paired-end reads), achieving a mean coverage depth of >100× across the exome. Raw sequencing data underwent standard preprocessing: adapter sequences were trimmed, and low-quality reads were filtered using Illumina’s bcl2fastq software. Cleaned reads were aligned to the GRCh37/hg19 human reference genome using BWA-MEM (v0.7.17). Post-alignment processing included duplicate marking, local realignment around insertions/deletions, and base quality score recalibration using the Genome Analysis Toolkit (GATK v4.1.8.1). Variant calling was performed using GATK HaplotypeCaller. Trio-based variant prioritization employed the following sequential filters: 1) Annotation using ANNOVAR, filtering against population allele frequencies [dbSNP, 1000 Genomes, Exome Aggregation Consortium (ExAC), Genome Aggregation Database (gnomAD)]; 2) Assessment of predicted functional impact using multiple algorithms (MutationTaster, SIFT, PolyPhen-2, PROVEAN, CADD, REVEL, and M-CAP) and evolutionary conservation scores (GERP++, phyloP, phastCons); 3) Classification according to the American College of Medical Genetics and Genomics/Association for Molecular Pathology (ACMG/AMP) guidelines [11]; 4) Correlation with known disease phenotypes using curated databases [Human Gene Mutation Database (HGMD), ClinVar, Online Mendelian Inheritance in Man (OMIM)]. Candidate variants identified through this pipeline were validated by bidirectional Sanger sequencing using specific primers. For detailed experimental procedures, see the Supplementary Material.

### Protein structural analysis

Our analysis of the canonical human ATP2B1 isoform (UniProt ID: P20020) was based primarily on the high-resolution cryo-EM structure of a homologous P-type ATPase (PDB ID: 6A69) [12]. Subsequently, to introduce the specific variant, the atomic coordinates for c.2140A>C (p.Thr714Pro) were modeled by performing residue substitution within the UCSF ChimeraX software package (v1.7.1). This enabled comparative visualization and analysis of the wild-type (WT) and mutant protein conformations.

### Plasmid construction and site-directed mutagenesis

The functional experimental strategy, including the assessment of calcium homeostasis, was designed based on established methods for investigating *ATP2B1* variant pathogenicity [8]. The full-length coding sequence of wild-type (WT) human *ATP2B1* (RefSeq NM_001366521.1) was PCR-amplified from commercial human brain cDNA (BioCat GmbH, Heidelberg, Germany) using high-fidelity Phanta Max Super-Fidelity DNA Polymerase (#P505, Vazyme Biotech). The amplicon was subsequently fused in-frame to the N-terminus of the mScarlet-I fluorescent protein using the ClonExpress Ultra One Step Cloning Kit (#C115, Vazyme Biotech), generating the WT ATP2B1-mScarlet construct. The T714P mutant (c.2140A>C) was generated using the WT ATP2B1-mScarlet plasmid as a template and mutation-specific primers via overlap extension PCR. All constructs (WT and T714P) were directionally cloned into the mammalian expression vector pcDNA3.1(+) (Thermo Fisher Scientific) using NotI and EcoRI restriction sites. PCR products and restriction digests were electrophoresed on 1.5% agarose gels (#SH441-01, Biosharp) stained with GelRed Nucleic Acid Gel Stain (#41005, Biotium). Bands of the correct size were excised and purified using a Gel Extraction Kit (#DP209, Tiangen Biotech). Purified inserts were ligated into EcoRI/NotI-digested pcDNA3.1(+) vector using T4 DNA Ligase (New England Biolabs). Recombinant plasmids were transformed into chemically competent *E. coli* DH5α cells. Positive clones were selected on LB agar plates containing 100 μg/mL ampicillin and screened by colony PCR. Plasmid DNA from positive clones was purified (#DP103, Tiangen Biotech) and the entire ATP2B1 insert was validated by bidirectional Sanger sequencing (Tsingke Biotech).

### Cell culture and transient transfection

HEK293T cells (Shanghai Cell Bank of the Chinese Academy of Sciences) were cultured in Dulbecco’s Modified Eagle Medium (#11965092, DMEM; Gibco) supplemented with 10% fetal bovine serum (FBS; Gibco, 10099141) and 1% penicillin-streptomycin (#15140122, Gibco). Cells were maintained at 37 °C in a humidified incubator with 5% CO_2_. For transient transfection, cells were seeded onto poly-L-lysine-coated coverslips in 6-well plates or directly into plates and grown to 50–60% confluence. Transfection complexes were prepared by mixing 2 μg of plasmid DNA (WT ATP2B1-mScarlet, T714P ATP2B1-mScarlet, or empty pcDNA3.1 vector control) with 7 μL Lipofectamine 3000 reagent and 4 μL P3000 Enhancer Reagent (#L3000015, Thermo Fisher Scientific) in 250 μL Opti-MEM I Reduced Serum Medium (#31985070, Gibco). The mixture was incubated for 15 min at room temperature before adding dropwise to the cells. After 6 h of incubation, the transfection mixture was replaced with fresh complete growth medium. Cells were analyzed 48 h post-transfection.

### Subcellular localization analysis by confocal microscopy

At 48 h post-transfection, cells grown on coverslips were washed twice with phosphate-buffered saline (PBS) and fixed with 4% paraformaldehyde in PBS for 15 min at room temperature. After fixation, cells were washed three times with PBS and permeabilized with 0.1% Triton X-100 in PBS for 10 min. Nuclei were counterstained with 10 μg/mL Hoechst 33342 (#C1028, Beyotime Biotech) in PBS for 15 min at room temperature. Coverslips were mounted onto glass slides using ProLong Gold Antifade Mountant (#P36930, Thermo Fisher Scientific). Fluorescence imaging was performed using a Leica TCS SP8 confocal microscope equipped with a ×63 oil immersion objective (NA 1.4). Excitation/emission settings were optimized for mScarlet (excitation: 569 nm, emission: 593–620 nm) and Hoechst 33342 (excitation: 405 nm, emission: 410–480 nm). Z-stacks were acquired with a step size of 0.5 μm. Plasma membrane localization was quantified using ImageJ software (NIH). The EzColocalization plugin was used to generate line-scan intensity profiles across cell peripheries from maximum intensity projections. The ratio of plasma membrane fluorescence intensity (peak) to adjacent cytoplasmic fluorescence intensity (trough) was calculated for at least 30 cells per condition from three independent experiments.

### Measurement of intracellular calcium levels

This assay was performed to assess the impact of the *ATP2B1* variant on basal cytosolic calcium concentration, a key indicator of calcium homeostasis. The approach of measuring resting Ca^2+^ levels in transfected cells aligns with the functional validation methodology employed in prior studies of *ATP2B1*-related disorders [8]. Transfected HEK293T cells in 6-well plates were washed twice with pre-warmed PBS and loaded with 5 μM Fluo-4, AM (#KGE3103-1, KeyGEN BioTECH) dissolved in PBS containing 0.02% pluronic F-127 (#P3000MP, Thermo Fisher Scientific) for 30 min at 37 °C in the dark. After loading, cells were washed three times with PBS to remove excess dye and incubated in dye-free PBS for an additional 20 min at 37 °C to allow complete de-esterification of the AM ester. Cells were then gently trypsinized, resuspended in fresh PBS, and transferred to glass-bottom dishes for immediate imaging. Intracellular Ca^2+^ levels were quantified by measuring Fluo-4 fluorescence intensity using an Olympus IX83 fluorescence microscope equipped with a FITC filter set (excitation: 494 nm, emission: 516 nm) and a cooled CCD camera. Images were acquired using identical exposure times (200 ms) and gain settings to allow direct comparison. Baseline fluorescence intensity was measured in the absence of any stimulation for ≥100 individual cells per condition (WT, T714P, empty vector) from three independent biological replicates. To minimize photobleaching and dye leakage, cells were imaged rapidly, and exposure time was strictly limited. Fluorescence intensity values were background-subtracted using cell-free regions. Mean fluorescence intensity per cell was calculated.

### Statistical analysis

All experiments were performed with a minimum of three independent biological replicates, each consisting of technical triplicates where applicable. Data are presented as mean ± standard error of the mean (SEM). Statistical significance between two groups was determined using unpaired, two-tailed Student’s t-tests. Analyses were performed using GraphPad Prism software (v8.0). A p-value <0.05 was considered statistically significant. ^**^ *p* < 0.01, ^***^ *p* < 0.001.

## Results

### Patient and clinical evaluation

An 8-month-old female infant was the first child (G1P1) of non-consanguineous Chinese parents. She was delivered vaginally at 35^+3^ weeks of gestation following preterm premature rupture of membranes. Severe intrauterine growth restriction was evident [birth weight 2,260 g (<3rd percentile, Z-score −2.8), length 49 cm (<3rd percentile, Z-score −2.3)]. A comprehensive review of the perinatal records revealed no clinical or biochemical indicators of asphyxia. At 2 months of age, she developed acute-onset infantile spasms. The seizures manifested as 10–15 daily clusters, each cluster comprising 20–30 individual flexor-dominant events. Semiology included repetitive head-nodding (“salaam attacks”), bilateral shoulder elevation, asymmetric tonic limb posturing, and axial hyperextension, predominantly occurring during sleep-wake transitions. The infant exhibited consistent post-ictal irritability. Concurrently, a developmental plateau was observed. Furthermore, brain MRI revealed no evidence of structural abnormalities or patterns indicative of hypoxic-ischemic injury. Systemic investigations (ECG, abdominal ultrasound) were normal. Video-EEG monitoring demonstrated significant abnormalities: Interictally, there was diffuse background slowing (4–5 Hz) with multifocal epileptiform discharges. During wakefulness, bilateral occipital-dominant sharp waves showed a right-greater-left asymmetry. Sleep stages featured prominent midline spike-wave complexes (2.5–3 Hz). Growth parameters remained severely restricted at 8 months (weight 7.5 kg [<−2 SD], length 68 cm [<−2 SD]). Gesell Developmental Schedules assessment at 8 months confirmed global impairment across all domains (Table 1): Adaptive skills (Developmental Quotient [DQ] = 70) revealed deficits in object permanence and cause-effect understanding; Gross motor function (DQ = 68) manifested as an inability to sit independently; Fine motor abilities (DQ = 65) showed an immature palmar grasp without object transfer; Personal-social interaction (DQ = 74) demonstrated poor eye contact and limited social referencing; Language development (DQ = 69) was restricted to reduplicated babbling (“ba-ba”, “ma-ma”) without communicative gestures. This constellation of findings prompted comprehensive genetic investigation: refractory epileptic spasms, persistent microsomatism, and developmental arrest in the absence of structural lesions.

**TABLE 1:** Developmental assessment of the proband using Gesell Developmental Schedules at 8 months.

Domain	Developmental quotient (DQ)	Severity classification
Adaptive	70	Mild delay
Gross motor	68	Mild delay
Fine motor	65	Mild delay
Personal-social	74	Mild delay
Language	69	Mild delay

### Identification of a novel *de novo ATP2B1* variant

**FIGURE 1:**
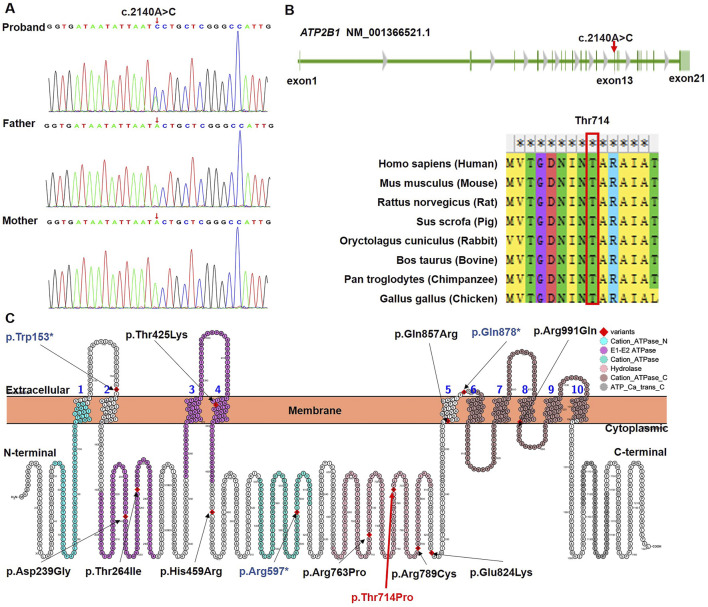
Genetic characterization of the novel *ATP2B1* variant. **(A)** Sanger sequencing chromatograms confirming the *de novo* heterozygous *ATP2B1* c.2140A>C variant (arrow) in the proband and its absence in both parents. **(B)** Evolutionary conservation analysis of Thr714 residue across diverse species (analyzed using MEGA12), highlighting its critical conservation. The mutated residue is indicated by the red box. **(C)** Schematic representation of the ATP2B1 protein domain architecture (generated using Protter) depicting the novel p.Thr714Pro variant (red) alongside the 12 previously reported MRD66-associated *de novo* variants (black: missense; dark blue: nonsense). Functional domains are annotated: 10 transmembrane helices (TM1-10), N-terminal cation transport ATPase region, catalytic E1-E2 ATPase, P-type cation transport ATPase domain, haloacid dehalogenase-like hydrolase (location of Thr714), C-terminal cation transport ATPase region, and C-terminal calmodulin-binding autoinhibitory region.

Trio-based WES identified a heterozygous missense variant in *ATP2B1* (NM_001366521.1: c.2140A>C) in the proband, resulting in the substitution of proline for threonine at amino acid position 714 (p.Thr714Pro; chr12:90005077, GRCh37/hg19). Bidirectional Sanger sequencing confirmed that this variant was absent in both unaffected parents (Figure 1A; PS2) and in major population databases (gnomAD v4.1.0, dbSNP v155, 1000 Genomes Phase 3, ExAC v0.3.1; PM2_Supporting). *ATP2B1* exhibits strong intolerance to missense variation, as evidenced by a high gnomAD missense Z-score (7.01; constraint threshold ≥3.09; PP2). Computational prediction tools unanimously supported a deleterious effect: Provean (−5.63), SIFT (0.0), PolyPhen-2 (1.0), MutationTaster (1), M-CAP (0.52), REVEL (0.96), CADD (27.5) (PP3). Thr714 residue is highly conserved across diverse species (Figure 1B). This variant represents a novel addition to the 12 previously reported *ATP2B1* variants associated with MRD66 (Figure 1C; Table 2). No other pathogenic variants or copy-number variations explaining the proband’s phenotype were detected. Based on ACMG/AMP guidelines (PS2 + PM2_Supporting + PP2 + PP3), the *ATP2B1* c.2140A>C (p.Thr714Pro) variant was classified as Likely Pathogenic.

**TABLE 2:** Clinical features of the proband and previously reported patients with *ATP2B1* vriants (NM_001366521.1).

Patient (Ref.)	Variant	Inheritance	Age	Sex	DD/ID	Epilepsy	Growth	Brain MRI	Facial/Skeletal features	Additional features	Functional evidence
This study	c.2140A>C (p.Thr714Pro)	*De novo*	8 ms	F	Mild DD	Infantile spasms	Ht/Wt SD <−2	Normal	Normal	Normal	↓ Ca^2+^ export, mislocalization
P1 [8]	c.716A>G (p.Asp239Gly)	*De novo*	6 ys	F	Moderate ID	Yes	Normal	NA	Facial dysmorphism, toe clinodactyly	Hypotonia, secundum atrial septal defect	↓ Ca^2+^ export, mislocalization
P2 [8]	c.791C>T (p.Thr264Ile)	*De novo*	8 ys	M	Mild ID	No	Normal	NA	Low-set ears	ASD, transposition of great vessels	↓ Ca^2+^ export
P3 [8]	c.1274C>A (p.Thr425Lys)	*De novo*	9 ys	M	Mild ID	Abnormal EEG	Normal	Abnormal	Sparse hair	Cerebral cavernous malformation	↓ Ca^2+^ export, mislocalization
P4 [8]	c.1376A>G (p.His459Arg)	*De novo*	17 ys	M	Mild ID	Yes	Normal	NA	Marfanoid habitus, arachnodactyly	ASD, scoliosis, hypermobile thumb	↓ Ca^2+^ export
P5 [8]	c.2288G>C (p.Arg763Pro)	*De novo*	21 ys	M	Mild ID	No	Normal	NA	Facial dysmorphism	ASD	↓ Ca^2+^ export, mislocalization
P6 [8]	c.2365C>T (p.Arg789Cys)	*De novo*	3 ys	M	ID	Yes	Normal	NA	Facial dysmorphism, hypotonia	Sleeping difficulties	↓ Ca^2+^ export
P7 [8]	c.2470G>A (p.Glu824Lys)	*De novo*	22 ys	F	Moderate ID	Yes	Normal	NA	–	Compulsive behavior	↓ Ca^2+^ export, mislocalization
P8 [8]	c.2570A>G (p.Gln857Arg)	*De novo*	3 ys	F	ID	No	Normal	NA	Brachycephaly, facial dysmorphism, 5th-finger clinodactyly	Normal	↓ Ca^2+^ export, mislocalization
P9 [8]	c.2972G>A (p.Arg991Gln)	Unknown	51 ys	M	Mild ID	No	Normal	NA	Marfanoid habitus, facial dysmorphism	ASD, aortic dilation, pectus carinatum, scoliosis	↓ Ca^2+^ export, mislocalization
P10 [8]	c.2632C>T (p.Gln878*)	*De novo*	6 ys	M	Mild ID	No	Normal	NA	Facial dysmorphism	Hyperactivity	Nonsense (NMD predicted)
P11 [8]	c.458G>A (p.Trp153*)	Unknown	6 ys	F	Moderate ID	No	Short stature	NA	–	NA	Nonsense (NMD predicted)
P12 [8]	c.1789C>T (p.Arg597*)	Unknown	5 ys	M	ID	Infantile spasms	Short stature	NA	Pectus excavatum, plagiocephaly	ASD	Nonsense (NMD predicted)
[13]	c.2938G>T (p.Val980Leu) c.3060 + 2T>G	Compound heterozygous	14 ys	M	Moderate ID (WISC:50)	No	Short stature	PVNH	Distinct craniofacial gestalt, microretrognathia, cleft palate	PVNH, brachydactyly, syndactyly, hypoparathyroidism	↓ ATP2B1 protein, ↓ Ca^2+^ export; splicing defect (NMD)

*Abbreviations*: DD: developmental delay, ID: intellectual disability, ASD: autism spectrum disorder, NA: Not Available/Absent, PVNH: periventricular nodular heterotopia, WISC: wechsler intelligence scale for children, SD: standard deviation, EEG: electroencephalogram, MRI: magnetic resonance imaging, mo: months, yrs: years, Ht: Height, Wt: Weight, TGV: transposition of great vessels, NMD: Nonsense-Mediated Decay.

### Structural impact of the p.Thr714Pro variant

**FIGURE 2:**
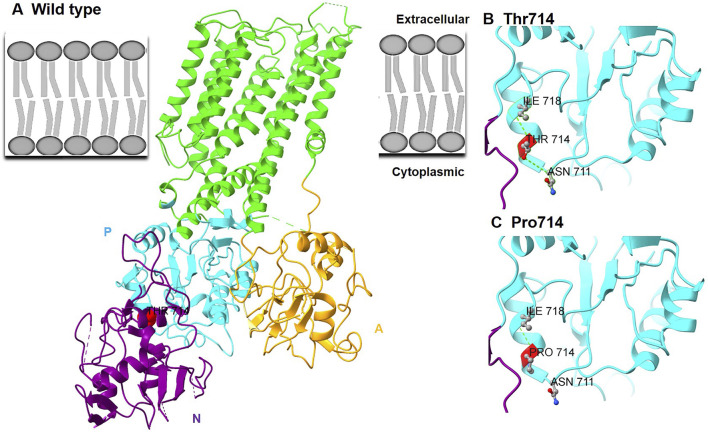
Structural impact of the p.Thr714Pro variant in *ATP2B1*. **(A)** 3D structure of wild-type ATP2B1 (based on PDB: 6A69): Phosphorylation (P) domain (blue), nucleotide-binding (N) domain (purple), and actuator (A) domain (gold). Thr714 (highlighted as red spheres) resides at the P-N domain interface within the P domain. The approximate location of the plasma membrane is indicated. **(B)** Close-up view of wild-type interactions involving Thr714: Hydrogen bonds (green dashed lines) between the Thr714 sidechain hydroxyl and the backbone carbonyl of Asn711 and the backbone amide of Ile718 stabilize the local α-helix. **(C)** Mutant conformation (p.Thr714Pro): Substitution with proline eliminates the hydroxyl group, preventing hydrogen bond formation with Asn711.

Homology modeling based on the human ATP2B1 cryo-EM structure (PDB: 6A69) positioned Thr714 within the P domain, specifically on a solvent-exposed α-helix (Figure 2A). This residue participates in essential hydrogen bonding networks that stabilize the local P-domain structure: the sidechain hydroxyl group of Thr714 forms a hydrogen bond with the backbone carbonyl oxygen of Asn711 and another with the backbone amide nitrogen of Ile718 (Figure 2B). The substitution of proline for threonine at position 714 induces significant structural perturbations: 1) Steric Incompatibility: Proline’s rigid cyclic side chain generates steric clashes (minimum atomic distance ∼1.8 Å) with the side chain of the adjacent Asn711 residue; 2) Loss of Critical Hydrogen Bonds: The absence of the hydroxyl group in proline ablates the hydrogen bonds normally formed between Thr714 and Asn711 (Figure 2C). These structural modifications destabilize the P domain interface required for calcium-dependent conformational changes during pump activation.

### The p.Thr714Pro variant disrupts plasma membrane trafficking of ATP2B1

**FIGURE 3:**
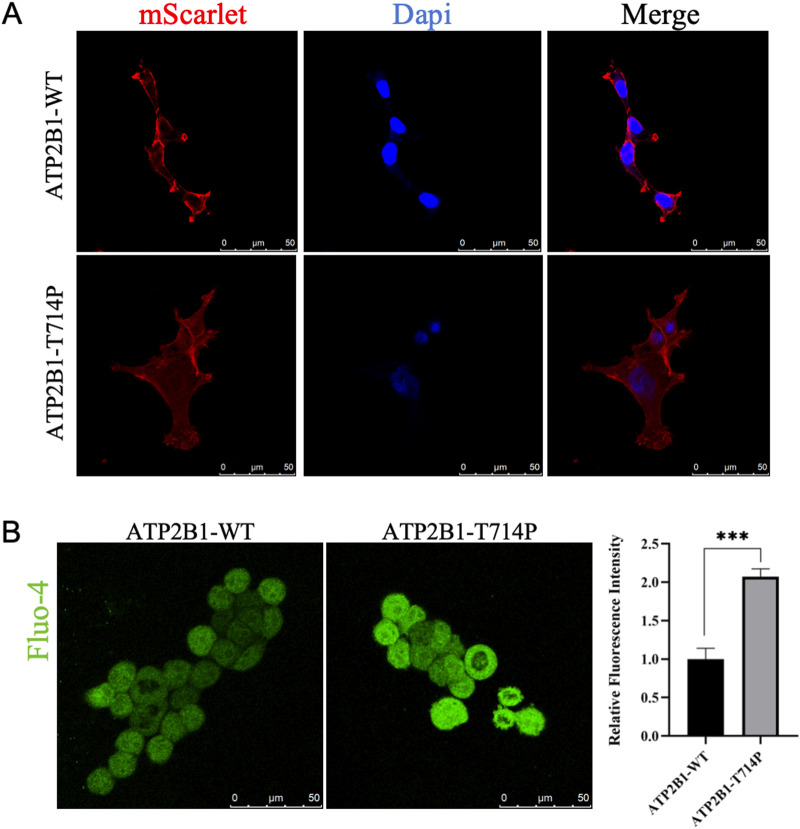
Functional Validation of the p.Thr714Pro Variant. **(A)** Confocal microscopy images of HEK293T cells expressing mScarlet-tagged ATP2B1 constructs (red). Nuclei are counterstained with Hoechst 33342 (blue). ATP2B1-WT-mScarlet localizes predominantly to the plasma membrane. ATP2B1-T714P-mScarlet shows diffuse cytoplasmic distribution with minimal plasma membrane signal. Scale bars: 20 μm. Insets show ×3 magnified regions of interest. **(B)** Quantification of basal intracellular Ca^2+^ levels using Fluo-4 AM fluorescence intensity. Data represent mean fluorescence intensity ±SEM from three independent experiments (n ≥ 100 cells per condition per experiment). ^***^ *p* < 0.001 vs. WT ATP2B1 (unpaired t-test). EV: Empty Vector control.

Confocal microscopy analysis revealed stark differences in the subcellular distribution of WT and mutant ATP2B1-mScarlet fusion proteins expressed in HEK293T cells (Figure 3A). ATP2B1-WT-mScarlet exhibited robust localization primarily at the plasma membrane, characterized by sharp, continuous fluorescence outlining the cellular periphery. In stark contrast, the T714P mutant displayed a predominantly diffuse cytoplasmic distribution, with significantly diminished fluorescence signal at the plasma membrane. Line-scan intensity profile analysis across cell boundaries confirmed a substantial reduction in membrane-associated fluorescence intensity for the T714P mutant (peak/trough ratio = 1.2 ± 0.3, mean ± SEM) compared to the WT construct (4.3 ± 0.5) (*p* < 0.001), representing a 72.3% decrease. This mislocalization phenotype demonstrates that the p.Thr714Pro substitution severely impairs the trafficking of PMCA1 to its functional site on the plasma membrane.

### Impaired calcium homeostasis in cells expressing the p.Thr714Pro variant

Given the critical role of PMCA1 in calcium extrusion, we assessed the functional consequence of mislocalization by measuring resting cytosolic Ca^2+^ levels using Fluo-4 AM fluorescence imaging (Figure 3B). Cells expressing the ATP2B1-T714P mutant exhibited profoundly elevated basal intracellular Ca^2+^ levels. The mean Fluo-4 fluorescence intensity was 2.07-fold higher in mutant-expressing cells compared to cells expressing WT ATP2B1 (*p* < 0.001). Cells transfected with the empty vector control exhibited fluorescence intensity between that of WT and T714P-expressing cells, and the differences were statistically significant compared to both (WT vs. empty vector, *p* < 0.01; T714P vs. empty vector, *p* < 0.001). This aberrant accumulation of cytosolic calcium provides direct functional evidence that the p.Thr714Pro variant compromises the Ca^2+^ extrusion capacity of PMCA1, leading to calcium dyshomeostasis. While this single-timepoint measurement reflects the net outcome of calcium influx, buffering, and efflux, a significant elevation in basal cytosolic Ca^2+^ is a consistent functional hallmark of pathogenic *ATP2B1* missense variants [8], indicating disrupted calcium homeostasis.

## Discussion

We identified a novel *de novo ATP2B1* missense variant, c.2140A>C (p.Thr714Pro), in a Chinese infant presenting with the core features of MRD66, including early-onset seizures and severe global developmental delay. Functional characterization demonstrates that the variant impairs *ATP2B1* function by causing its mislocalization away from the plasma membrane. This reduction in properly localized protein leads to a consequent loss of calcium efflux capacity. These findings establish that p.Thr714Pro causes haploinsufficiency via combined mislocalization and loss of catalytic activity, resulting in elevated cytosolic calcium (Ca^2+^), neuronal hyperexcitability, and the observed phenotype of global developmental delay with infantile spasms.

*ATP2B1* encodes the plasma membrane Ca^2+^-ATPase isoform 1 (PMCA1), a ubiquitously expressed P-type ATPase. PMCA1 is characterized by large cytoplasmic domains [Actuator (A), Nucleotide-binding (N), Phosphorylation (P)] and ten transmembrane helices (M1-M10) that form the ion transport pathway, making it essential for maintaining intracellular Ca^2+^ homeostasis via Ca^2+^ extrusion [12, 14] (Figures 1C, 2A). Its critical role in development is underscored by embryonic lethality in homozygous *Atp2b1* knockout mice [15], whereas heterozygous knockouts exhibit systemic alterations such as hypertension and abnormal bone metabolism [16]. In neurodevelopment, PMCA1 is particularly vital. Murine studies show a surge in its expression during key events like neural tube closure and neuronal migration [17], and it is expressed in the developing cerebral cortex [18]. This pattern is consistent with the established role of spatially and temporally regulated Ca^2+^ signaling in coordinating neurogenesis, neuronal differentiation, and circuit formation. In humans, biallelic *ATP2B1* variants are associated with a neurodevelopmental malformation syndrome with primary hypoparathyroidism [13], whereas *de novo* variants are the predominant cause of MRD66, a disorder defined by neurodevelopmental delay [8].

Deleterious heterozygous *ATP2B1* variants were first linked to MRD66 by Rahimi et al., who identified 12 patients with GDD, variable ID (mild to severe), and behavioral abnormalities (including ASD in 42%); seizures (infantile spasms, myoclonic, absence) were present in 50% of cases [8]. Phenotypic heterogeneity was notable, ranging from ambulatory patients with mild learning deficits to severely affected non-ambulatory and nonverbal individuals. Our patient’s presentation aligns with these core features of MRD66, exhibiting GDD and early-onset infantile spasms. Notably, the pronounced and persistent growth retardation (height and weight consistently below −2 SD) observed in our case represents a more severe manifestation of this feature compared to most previously reported heterozygous cases, thereby expanding the known phenotypic spectrum of MRD66 (Table 2). This degree of growth delay, although variable across reported patients, underscores the potential role of PMCA1 dysfunction in somatic growth pathways beyond neurodevelopment. In contrast to patients with biallelic *ATP2B1* variants who often present with multi-system malformations [13], our patient lacked overt dysmorphic features, structural brain anomalies, or biochemical evidence of hypocalcemia, further supporting haploinsufficiency as the primary disease mechanism in heterozygous carriers.

Thr714 resides within the conserved TGES motif of the haloacid dehalogenase-like hydrolase (HAD) domain, which is integral to the dephosphorylation step of the catalytic cycle during Ca^2+^ transport. Notably, two other MRD66 variants within this catalytic domain (p.Arg763Pro, p.Arg789Cys) also cause loss-of-function [8], highlighting the functional sensitivity of this region. The severe functional deficit caused by p.Thr714Pro aligns perfectly with *ATP2B1* 's documented high intolerance to variation (gnomAD pLI = 1.0, missense Z = 5.29). Currently, only ∼12 pathogenic *de novo* heterozygous variants (missense [75%], with nonsense [25%]) are associated with MRD66, distributed throughout the gene without clear genotype-phenotype correlations [8]. The existence of asymptomatic heterozygous carriers (e.g., the mother carrying the c.3060 + 2T>G splicing variant reported by Yap et al. [13]) underscores that the pathogenicity of ATP2B1 variants is not determined by inheritance pattern alone, but by the extent of functional impairment. This observation supports the concept of a threshold effect, whereby clinical manifestations arise only when the resultant calcium dyshomeostasis exceeds a physiological compensatory capacity. Notably, some missense variants may exert a more profound dominant-negative or toxic gain-of-function effect compared to alleles leading to simple haploinsufficiency, thereby reaching this pathogenic threshold more readily. Altered Ca^2+^ dynamics could also disrupt the E/I balance, possibly involving modulation of purinergic signaling pathways (e.g., P2X7 receptors impacting GABAergic function) [19]. Our functional characterization demonstrates that the p.Thr714Pro variant impairs PMCA1 function through a dual mechanism: defective trafficking to the plasma membrane and consequent failure to maintain low resting cytosolic Ca^2+^. This mislocalization phenotype is reminiscent of several pathogenic missense variants reported by Rahimi et al. (e.g., p.Asp239Gly, p.Thr264Ile, p.Arg991Gln) [8]. The significant elevation of basal intracellular Ca^2+^ we observed is a consistent finding across all nine missense variants functionally tested in that study. Importantly, the p.Thr714Pro variant resides within the critical P-domain. Our structural modeling suggests it disrupts key hydrogen bonds (Thr714-Asn711), likely destabilizing the local structure. This provides a plausible molecular explanation for both the mislocalization (due to protein misfolding) and the loss of calcium transport activity. Combined, our genetic, cellular, and *in silico* data robustly support the pathogenicity of the novel p.Thr714Pro variant via a loss-of-function mechanism.

Our findings have immediate clinical implications. *ATP2B1* sequencing should be prioritized in infants presenting with a neurodevelopmental disorder with early-onset seizures, even in the absence of dysmorphism or overt systemic features. This notion aligns with the concept of a pathogenic threshold, where the severity of clinical presentation correlates with the degree of functional impairment [13]. Heterozygous variants, particularly those that cause Haploinsufficiency, are associated predominantly with neurodevelopmental deficits, while biallelic genotypes lead to multi-system malformations, including primary hypoparathyroidism, distinctive craniofacial and skeletal features, and periventricular heterotopia [13]. The central role of calcium dyshomeostasis in pathogenesis supports the careful consideration of calcium channel modulators (e.g., ethosuximide for absence seizures, nimodipine) as adjunctive therapy for seizure control, although efficacy specifically in *ATP2B1*-related epilepsy requires further study. Early intensive developmental intervention is critical. Our patient’s severe growth restriction necessitates proactive management, including endocrine evaluation, quarterly anthropometric monitoring, high-calorie nutritional supplementation, and surveillance of serum calcium and parathyroid hormone levels, even in the absence of current hypocalcemia [13]. Proactive neurodevelopmental support is warranted, potentially including the Early Start Denver Model for ASD prevention given the high co-occurrence [8], and consideration of the ketogenic diet, which demonstrates ∼40% efficacy in infantile spasms, potentially via ketone-mediated reduction of neuronal excitability and attenuation of P2X7 receptor-mediated neuroinflammation [19]. Prognostically, uncontrolled infantile spasms persisting beyond 12 months confer a >70% risk of significant cognitive impairment; our patient may progress to moderate ID, particularly given the persistent background EEG abnormalities. Study limitations include reliance on the HEK293T cell model; future studies utilizing neuronal models (e.g., patient-derived iPSCs differentiated into neurons) and longitudinal natural history studies of larger *ATP2B1*-variant cohorts are essential to fully elucidate pathomechanisms, define genotype-phenotype correlations, and optimize clinical management strategies.

This study has several limitations. First, the calcium imaging assay measured basal Ca^2+^ levels rather than dynamically quantifying *ATP2B1*-mediated extrusion kinetics. While elevated resting Ca^2+^ is a validated indicator of *ATP2B1* dysfunction [8], future studies employing time-resolved efflux assays (e.g., measuring the exponential decline of Ca^2+^ after a load) would provide more direct insight into pump activity. Second, our functional analysis was conducted in a heterologous system (HEK293T cells). Although this model is well-established for the initial characterization of ion transporter variants, studies using patient-derived neurons would more accurately reflect the pathomechanism in the relevant cell type. Finally, the clinical cohort of individuals with pathogenic *ATP2B1* variants remains small. Larger studies are needed to establish definitive genotype-phenotype correlations and the full clinical spectrum of MRD66.

In conclusion, we identified a novel *de novo ATP2B1* p.Thr714Pro variant causing MRD66 through a dual-stage pathomechanism: defective plasma membrane trafficking and intrinsic impairment of catalytic function, culminating in calcium dysregulation. This case significantly expands the phenotypic spectrum of MRD66 by demonstrating the earliest seizure onset reported to date and reinforces haploinsufficiency as the primary mechanism underlying heterozygous *ATP2B1* variants. Our integrated genetic, structural, and functional findings underscore the indispensable role of PMCA1 in neurodevelopment and calcium homeostasis, providing a foundation for developing targeted therapeutic strategies aimed at mitigating calcium dyshomeostasis in this disorder.

## Data Availability

The data generated in this study are available from the corresponding author upon reasonable request. The variant data from this study have been submitted to the ClinVar database (Submission ID: SUB15908833).
