# Supplementary material for: A novel de novo ATP2B1 variant causes autosomal dominant intellectual developmental disorder 66 by disrupting calcium homeostasis via impaired membrane trafficking
Source: Exp Biol Med (Maywood). 2026 Mar 3;251:10834. doi: 10.3389/ebm.2026.10834 (PMC12992144; doi:10.3389/ebm.2026.10834)
Supplement: Supplementary file 1 [file Supplementaryfile1.docx]

Supplemental Methods

**Sanger sequence**

Based on the *ATP2B1* gene sequence from the NCBI GenBank database, specific primers were designed using Primer5.0 software. For the c.2140A>C variant, the forward primer (F: 5′-TATTTCATGCATGGTGCCTAACAC-3′) and reverse primer (R: 5′-TAAGTCTGGCAGTGGAATTGGAAA-3′) were designed, with an amplification product length of 498 bp. PCR reactions were performed using the KAPA2G Robust HotStart PCR Kit (KAPA Biosystems). The 25 µl reaction system contained: 5 µl of 5× Buffer A, 0.5 µl of 10 mM dNTPs, 1.25 µl each of 10 µM forward/reverse primers, 1 µl of template DNA, 0.1 µl of DNA polymerase, and PCR-grade water to make up the final volume. The amplification program was run on a Hema 9600 PCR Thermal Cycler (Zhuhai Hema): pre-denaturation at 95℃ for 3 minutes; 30 cycles of denaturation at 95℃ for 30 seconds, annealing at 60℃ for 30 seconds, and extension at 72℃ for 30 seconds; and a final extension at 72℃ for 1 minute. After verification of amplification products by 1.5% agarose gel electrophoresis, bidirectional sequencing was conducted using an ABI 3730XL Sequencer (Applied Biosystems). Raw data were aligned with the reference sequence via DNASTAR software (Lasergene v7.1).

**Plasmid Construction for ATP2B1 Wild-Type and T714P Mutant Fused with mScarlet**

The coding sequences (CDS) of human wild-type ATP2B1 and the T714P mutant (c.2140A>C, NM_001366521) were fused in-frame to the N-terminus of the mScarlet red fluorescent protein gene and cloned into the pcDNA3.1 vector using the NotI and EcoRI restriction sites. All PCR amplifications were performed using a TC-96 PCR Thermal Cycler (Bioer Technology) with Phanta Max Super-Fidelity DNA Polymerase (Vazyme Biotech, P505) to ensure high fidelity. Primer sequences for seamless cloning and site-directed mutagenesis are listed in the Supplementary Information. The ClonExpress Ultra One Step Cloning Kit (Vazyme Biotech, C115) was used for all recombination reactions. PCR products and enzyme-digested vectors were purified using the Agarose Gel DNA Extraction Kit (Tiangen Biotech, DP209) following electrophoresis on a 1.5% agarose gel prepared with Agarose (Beijing Biomarker, SH441-01) and stained with GelRed Nucleic Acid Gel Stain (Biotium, 41005). The DYCP-31DN Agarose Gel Electrophoresis System (Beijing Liuyi Biotechnology) was used for analysis. Restriction digestion of the pcDNA3.1 vector was performed using EcoRI (#R0101V) and NotI (#R0189V) (New England Biolabs). The ligation products were transformed into competent E. coli cells. Single colonies were selected after overnight culture at 37°C in a Bacterial Incubator (Shanghai Lichen Technology, HN-60BS) and sent for Sanger sequencing (Qingke Biotechnology, Nanjing).

**Plasmid Preparation**

Plasmid DNA was extracted from bacterial cultures using an Endo-Free Plasmid Midiprep Kit (Cowin Biotech, CW2105S) according to the manufacturer's protocol. Bacterial pellets were lysed, and endotoxins were removed via filtration through the provided column. DNA was precipitated, washed, and eluted in Endo-Free Elution Buffer. Concentrations were measured spectrophotometrically. Purified plasmids were stored at -80°C in an Ultra-Low Temperature Freezer (Anhui Zhongke Duling, MDF-86V340E).

**Cell Culture and Transfection**

HEK293T cells were maintained in a humidified CO₂ Cell Culture Incubator (Thermo Fisher, THERMO 3111) at 37°C with 5% CO₂. For transfection, cells were seeded in 6-well plates. When cell density reached 50-60%, they were transfected using Lipofectamine 3000 Reagent (Thermo Fisher). Briefly, for each well, 2 µg of plasmid DNA was mixed with 4 µL of P3000™ reagent in 125 µL of Opti-MEM™ medium. Separately, 7 µL of Lipofectamine 3000 was diluted in 125 µL of Opti-MEM™. After 5-minute incubation at room temperature, the two solutions were combined, incubated for 15 minutes to form complexes, and added dropwise to the cells. All cell culture procedures involving open vessels were performed under sterile conditions inside a SW-CJ-2FD Clean Bench (Shanghai Boxun). The culture medium was replaced 6 hours post-transfection.

**Fluorescence Microscopy for Protein Localization**

At 48 hours post-transfection, cells were stained with 5 µg/mL Hoechst 33342 for 15 minutes at 37°C to label nuclei. After washing with PBS, cells were imaged live using a SOPTOP-XD Fluorescence Inverted Microscope (Sunny Optical Technology) equipped with appropriate filter sets for mScarlet (red fluorescence) and Hoechst (blue fluorescence). Images were captured to assess the subcellular localization of the ATP2B1-mScarlet fusion proteins.

**Intracellular Ca²⁺ Measurement**

Intracellular Ca²⁺ levels were measured using the calcium-sensitive fluorescent probe Fluo-4, AM (KeyGEN BioTECH, KGE3103-1). At 48 hours post-transfection, HEK293T cells were washed with PBS and loaded with 5 µM Fluo-4, AM in culture medium at 37°C for 45 minutes. Cells were then washed three times with PBS to remove excess dye. Fluorescence was immediately detected using the fluorescence microscope with excitation/emission settings of approximately 490/516 nm. The relative fluorescence intensity from multiple random fields was quantified and used as an indicator of relative cytosolic Ca²⁺ concentration.
